# Regulation of granin neuropeptide gene expression in human brain during development

**DOI:** 10.3389/fnmol.2025.1666795

**Published:** 2025-12-02

**Authors:** Laura L. Demsey, Sonia Podvin, Vivian Hook

**Affiliations:** 1 Skaggs School of Pharmacy and Pharmaceutical Sciences, University of California, San Diego, La Jolla, CA, United States; 2 Department of Neurosciences, School of Medicine, University of California, San Diego, La Jolla, CA, United States; 3 Department of Pharmacology, School of Medicine, University of California, San Diego, La Jolla, CA, United States

**Keywords:** granins, neuropeptides, neurotransmitters, genes, human brain, development, VGF, CHGA

## Abstract

The granin gene family of neuropeptides functions as peptide neurotransmitters in the brain for the regulation of neural functions that regulate behaviors. Granins are involved in regulating cognition, memory, depression, aggression, stress, energy expenditure, inflammation, and related. Development of the human brain involves formation of synapses and their spectrum of neurotransmitters to establish neural connections that are required for brain functions. Therefore, the goal of this study was to analyze the gene expression profiles of the granin neurotransmitter genes during human brain development at prenatal, infancy, childhood, adolescence, and adult stages. Granin gene expression in brain development was assessed by quantitative RNA sequencing data from the Allen Human Brain Atlas resource. *VGF* (neurosecretory protein VGF) expression was significantly increased during development during the prenatal to childhood through adult stages in the anterior cingulate cortex, dorsolateral prefrontal cortex, inferolateral temporal cortex, orbital frontal cortex, posteroventral parietal cortex, primary somatosensory cortex, and primary visual cortex regions. *SCG2* (secretogranin 2) expression was also significantly increased from prenatal to infancy through adult stages in anterior cingulate cortex, dorsolateral prefrontal cortex, inferolateral temporal cortex, orbital frontal cortex, posterior superior temporal cortex, posteroventral parietal cortex, primary somatosensory cortex, and primary visual cortex. A modest number of brain regions showed increased *CHGA*, *CHGB*, and *SCG3* expression in the postnatal periods compared to the prenatal periods. Further, the *SCG5, PCSK1N, and GNAS* genes displayed minimal changes throughout development. Overall, these results demonstrate developmental upregulation of *VGF* and *SCG2* genes, with lesser upregulation of *CHGA*, *CHGB*, and *SCG3* genes, and almost no changes in *SCG5*, *PCSK1N*, and *GNAS* genes during development. These findings illustrate the differential regulation of granin genes during human brain development.

## Introduction

The granin family of neuropeptides function as peptide neurotransmitters for cell–cell signaling in the brain that is essential for the regulation of brain functions, including memory, depression, aggression, stress, energy utilization, inflammation, and related. The granin gene family is composed of chromogranin A (*CHGA*), chromogranin B (*CHGB*), secretogranin II (*SCG2*, also known as chromogranin C), secretogranin III (*SCG3*, also known as 1B1075), secretogranin V (*SCG5*, also known as 7B2), and *GNAS* (*SCG6*, also known as secretogranin VI and NESP55), proSAAS (also known as PCSK1N), and *VGF* (also known as secretogranin VII) (Taupenot et al., 2003; Bartolomucci et al., 2011; Troger et al., 2017; Quinn et al., 2023; Weiss et al., 2000).

Granins are produced as neuropeptide precursor proteins that are routed to dense core secretory vesicles (DCSV) where such precursors undergo proteolytic processing to generate neuropeptides that are released to mediate cell–cell signaling in neurotransmission (Hook et al., 2018). The granins also facilitate DCSV biogenesis that is required for neuropeptide production (Bartolomucci et al., 2011). The secreted brain neuropeptides reach the cerebrospinal fluid (CSF) where neuropeptides have been studied as biomarkers of brain health and neurological disease conditions (Higginbotham et al., 2020; Haque et al., 2023; Quinn et al., 2023).

Regulation of granin neuropeptides in brain disorders has been found to occur at diverse ages from young to adult years, shown by dysregulation of granins in schizophrenia (SZ) and traumatic brain injury (TBI) that occur at young through adult ages, and in Alzheimer’s disease (AD) and Parkinson’s disease (PD) that occur in older adults. In human SZ individuals, CHGB neuropeptides are reduced in brain hippocampus (Nowakowski et al., 2002); in SZ patient-derived induced neurons (iN) CHGB neuropeptides are reduced compared to control neurons (Podvin et al., 2024). In TBI mice, SCG2 was found to mediate blood–brain barrier dysfunction (Lin et al., 2024). In human AD brain and cerebrospinal fluid (CSF), VGF and CHGA neuropeptides are downregulated and upregulated, respectively, compared to control brains (Higginbotham et al., 2020; Podvin et al., 2022; Quinn et al., 2023; Haque et al., 2023); moreover, reduced VGF neuropeptides correlate with cognitive impairment (Haque et al., 2023; Higginbotham et al., 2020). In PD subjects, granins were generally reduced in CSF (Rotunno et al., 2020).

Animal studies of granin gene knockout have demonstrated roles for granins in memory, depression, energy expenditure, tauopathy, angiogenesis, and related (Lin et al., 2015; Watson et al., 2009; Pereda et al., 2015; Jati et al., 2025; Dai et al., 2022). In AD animal models, *VGF* gene ablation impaired memory function (Lin et al., 2015), and knockout of the *CHGA* gene resulted in improved cognitive function and amelioration of tau neuropathology (Jati et al., 2025). VGF has been shown to regulate energy expenditure and fat storage in *VGF* gene knockout mice (Watson et al., 2009). Mice lacking the *CHGB* gene exhibit increased aggression and depressive behaviors (Pereda et al., 2015). Deletion of the *SCG3* gene resulted in reduced severity of pathological angiogenesis in oxygen-induced retinopathy (OIR) (Dai et al., 2022).

Development of the human brain involves formation of the spectrum of granin neurotransmitters to establish neural signaling required for brain functions. The regulation of granins in brain disorders at young to adult ages raises the question of their developmental regulation of granin genes in human brain. Therefore, the goal of this study was to investigate the granin gene expression profiles in human brain development during the stages of the prenatal period to infancy, childhood, adolescence, and adult stages. Evaluation of granin gene expression in 16 human brain regions during development was achieved by analyzing quantitative Ref-seq data from the Allen Human Brain Atlas resource (Hawrylycz et al., 2012; Sunkin et al., 2013). Results illustrated differential upregulation of granin genes during human brain development, with VGF and SCG2 displaying the greatest prevalence of development upregulation among the brain regions studied.

## Methods and procedures

### Developmental expression of granin genes in human brain regions from the Allen human brain atlas resource

The Allen Human Brain Atlas resource for gene expression data (Hawrylycz et al., 2012; Sunkin et al., 2013) was utilized for analysis of granin gene expression levels during human brain development in 16 human brain regions during prenatal, infancy, childhood, adolescence, and young adult age periods. These data were achieved by the human brain development project of the Allen Brain Atlas that provides RNA sequencing data for quantitative gene expression of the 8 granin genes assessed in this study.

The Allen Human Brain Atlas collected postmortem human brain tissues at ages of early pre-natal, late prenatal, infancy, childhood, adolescence, and young adult. Ages for each developmental period is shown in Table 1. The brain tissue collection included males and females, with subjects having backgrounds of European, Asian, African American, Hispanic, and mixed cultures. The number of brain samples from each brain region, per developmental period, consisted of 4 to 12 samples from different subjects (males and females) that were determined by the Allen Human Brain Atlas program. It will be desirable in future studies to use large sample sizes per group and comparisons. Negative selection criteria included chromosomal abnormalities, maternal drug use, brain lesions, positive testing for hepatitis B or C, HIV, and neurological disorders.

**Table 1:** Developmental stages studied for granin gene expression in human brain.

Developmental stage	Age range	Number of brain samples
Early Prenatal	8–18 weeks post-conception	12 (7 males, 4 females)
Late Prenatal	19–38 weeks post-conception	8 (3 males, 5 females)
Infancy	8–18 months	5 (4 males, 1 female)
Childhood	19 months to 11 years	7 (4 males, 3 females)
Adolescence	12–19 years	4 (2 males, 2 females)
Young Adult	20–40 years	6 (3 males, 3 females)

The age ranges for each development stage are indicated. For each stage, 16 brain regions were assessed for granin gene expression levels.

Sixteen brain regions were analyzed, consisting of the amygdaloid complex, anterior cingulate cortex, cerebellar cortex, dorsolateral prefrontal cortex, hippocampus, inferolateral temporal cortex, mediodorsal nucleus of thalamus, orbital frontal cortex, posterior superior temporal cortex, posteroventral parietal cortex, primary auditory cortex, primary motor cortex, primary somatosensory cortex, primary visual cortex, striatum, and ventrolateral prefrontal cortex. Study of these brain regions was selected by the Allen Human Brain Atlas program because their development is essential for critical brain functions that include cognition, memory, executive function, language, motor movement, vision, emotion, social behavior, and olfaction (Table 2).

**Table 2:** Behavioral functions of human brain regions investigated for granin gene expression.

Brain Regions	Behavioral Functions									
	Cognition	Memory	Executivefunction	Language	Motor	Vision	Emotion	Social behavior	Olfaction	References
(a) Amygdaloid complex										Phelps and LeDoux (2005); Šimić et al. (2021)
(b) Anterior Cingulate Cortex										Etkin et al. (2011); Shackman et al. (2011)
(c) Cerebellar Cortex										Kandel et al. (1991)
(d) Dorsolateral Prefrontal Cortex										Kandel et al. (1991); Miller and Cohen (2001)
(e) Hippocampus										Lisman et al. (2018); Moscovitch et al. (2016)
(f) Inferolateral Temporal Cortex										Patterson et al. (2007); Robinson and Rolls (2015)
(g) Mediodorsal Nucleus of Thalamus										Wolff and Halassa (2024); Kandel et al. (1991)
(h) Orbital Frontal Cortex										Rolls (2023)
(i) Posterior Superior Temporal Cortex										Redcay (2008); Kandel et al. (1991)
(j) Posteroventral Parietal Cortex										Squire et al. (2003); Sokolowski et al. (2023)
(k) Primary Auditory Cortex										Squire et al. (2003); Weinberger (2015)
(l) Primary Motor Cortex										Schieber (2001); Squire et al. (2003)
(m) Primary Somato-sensory Cortex										Squire et al. (2003)Kandel et al. (1991)
(n) Primary Visual Cortex										Boynton (2005); Kandel et al. (1991)
(o) Striatum										Squire et al. (2003); O'Doherty (2004)
(p) Ventrolateral Prefrontal Cortex										Badre and Wagner (2006); Blumenfeld and Ranganath (2007)

Gene expression data were acquired by RNA-Seq analyses for the 16 brain regions, as described by the Allen Human Brain Atlas resource (Shen et al., 2012). Briefly, tissues were subjected to RNA extraction and polyA+ RNA was isolated using polyT-RNA magnetic beads. The purified polyA+ RNAs were quantitated and spike-in RNA mixes were added to normalize gene expression across samples. The polyA+ RNA was subjected to cDNA synthesis using reverse transcriptase and DNA polymerase I, combined with adenylation at 3′-ends, for DNA sequencing by the Illumina Genome Analyzer Iix. RNA-Seq data provided gene expression data expressed as RPKM, reads per kilobase of exon model per million mapped reads. This RNA-Seq data was obtained from the Allen Brain Atlas resource^1^ for gene expression analysis of granin genes.

The average gene expression level for each granin gene (in units of RPKM) was calculated for the six developmental periods. In addition, the total granin gene expression, sum of the 8 granin genes, was calculated for each developmental period as the mean +/- standard error of the mean (s.e.m). Statistical analyses utilized the Kruskal-Wallis non-parametric test, the Dunn’s multiple comparison *post hoc* test, and two-way ANOVA followed by Tukey’s post hoc multiple comparisons test using GraphPad Prism. Statistical significance was assessed as *p* < 0.05.

## Results

### Strategy for granin gene expression analysis in human brain development by RNA-seq analyses

**Figure 1:**
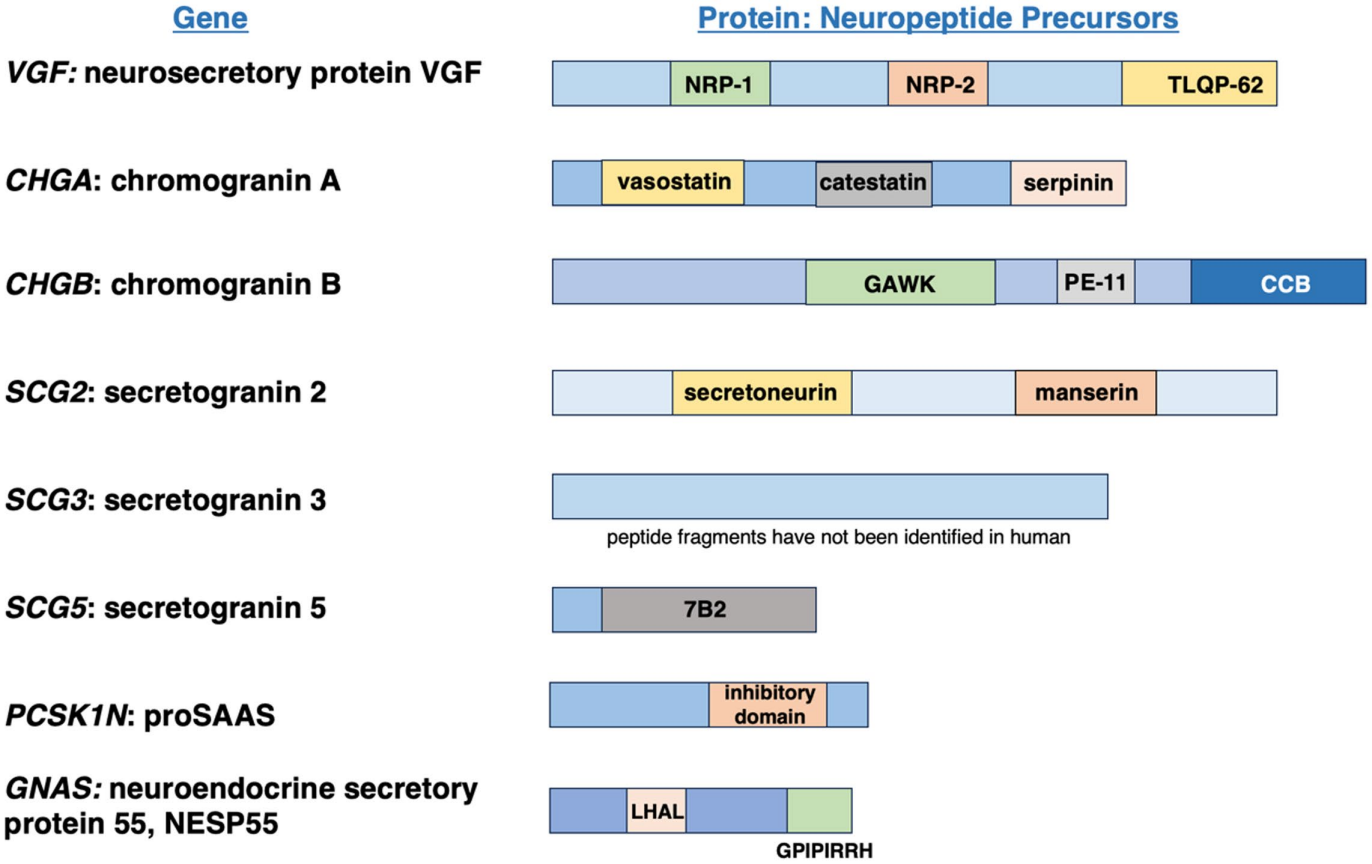
Granin genes encode neuropeptide transmitter precursor proteins. Granin gene expression in human brain during development to adult was conducted for the *VGF*, CHGA, *SCG2*, *SCG3*, *SCG5*, *PCSK1N* (proSAAS), and *GNAS* (NESP55) genes by RNA-seq analyses by the Allen Human Brain Atlas resource (Hawrylycz et al., 2012; Sunkin et al., 2013). Each granin gene encodes a neuropeptide precursor protein that undergoes proteolytic processing to generate small neuropeptide transmitters. Examples of neuropeptides generated from each translated granin protein are illustrated.

The granin genes of *VGF, CHGA, CHGB, SCG2, SCG3, SCG5, PCSK1N* (proSAAS), and *GNAS* encode proneuropeptide precursors that are converted to small neuropeptide transmitters by proteolysis (Figure 1). These granin genes are important for essential brain functions that include cognition, memory, executive function, language, movement, vision, emotion, social behavior, and olfaction (Table 2).

**Figure 2:**
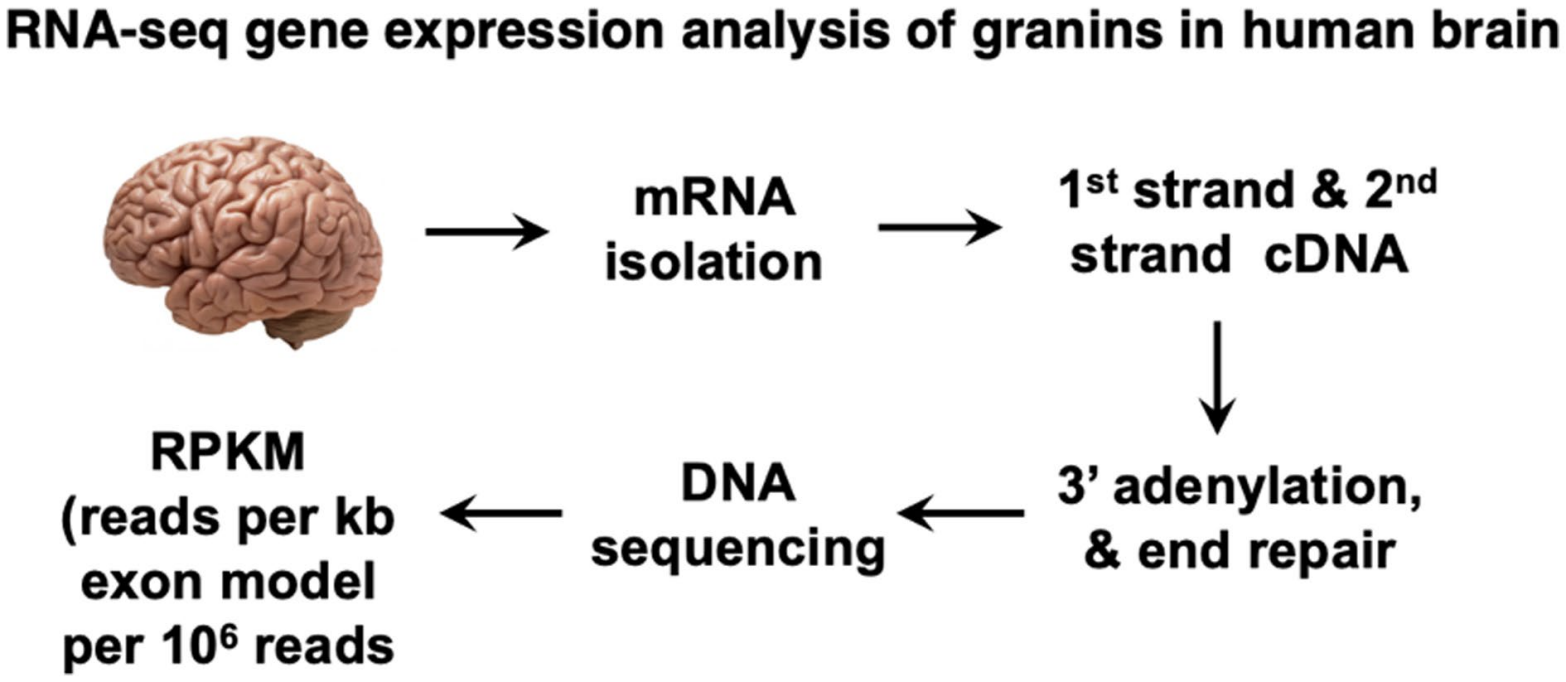
RNA-Seq expression analyses of granin genes in human brain. Expression of the *VGF*, *CHGA*, *CHGB*, *SCG2*, *SCG3*, *SCG5*, *PCSK1N* (proSAAS), and *GNAS* (NESP55) genes were assessed by RNA-Seq analysis of 16 brain regions during developmental periods of prenatal, infancy, childhood, adolescence, and young adult stages. RNA-Seq data was obtained from the open resource program of the Allen Human Brain Atlas. Gene expression was quantitated as RPKM (reads per kilobase of exon model per million mapped reads).

The expression levels of the granin genes *VGF, CHGA, CHGB, SCG2, SCG3, SCG5, PCSK1N* (proSAAS), and *GNAS* (Supplementary Table S1) in human brain were assessed during development from prenatal, to infancy, childhood, adolescence, and adult stages (Table 1) by RNA-seq analysis of 16 human brain regions (Figure 2). The data provided an extensive assessment of the 8 granin genes during development of the human brain.

### Total granin gene expression levels during development in human brain regions

**Figure 3:**
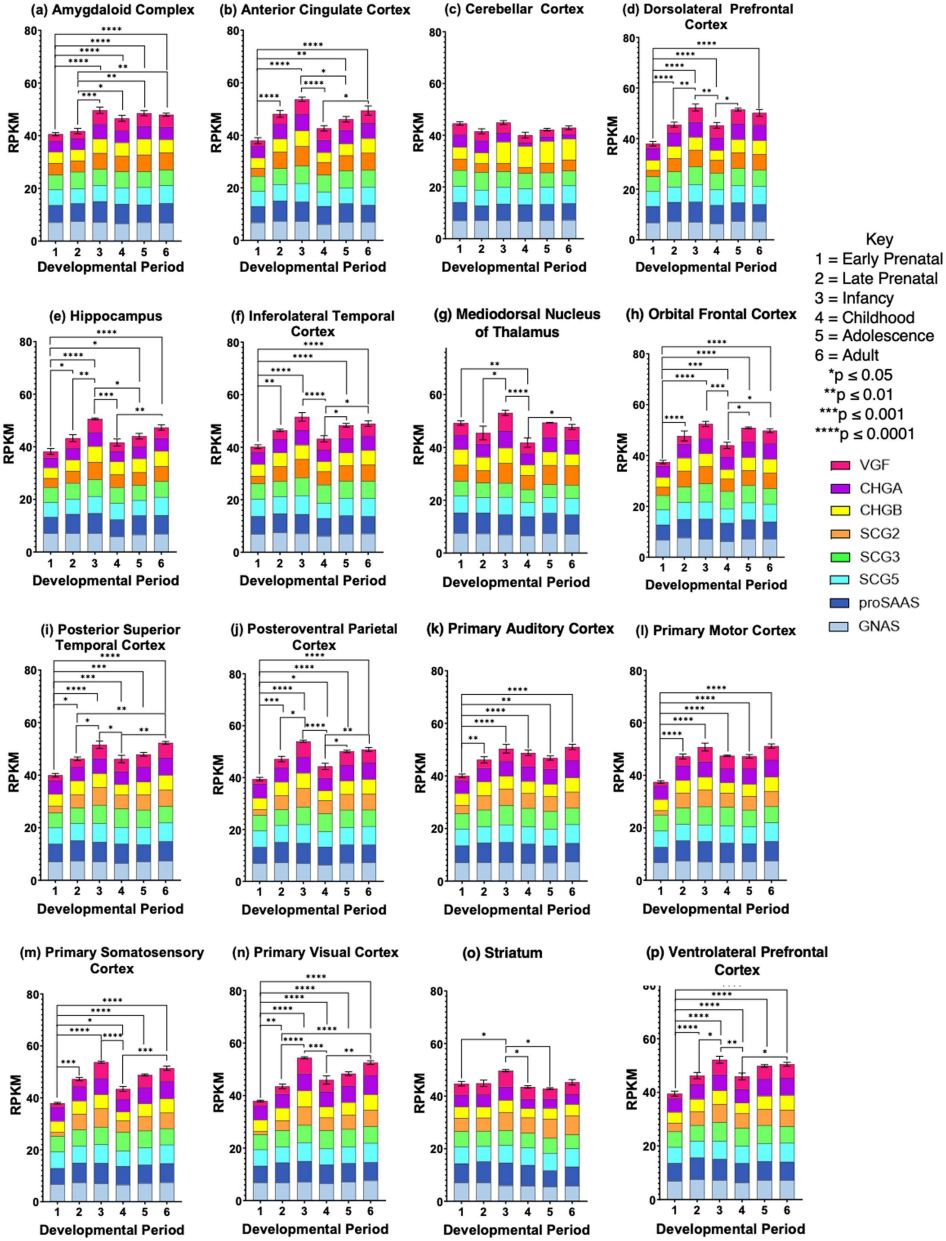
Total expression of granin genes during development in human brain. Total granin expression is expressed as the sum of the quantitative expression values for the 8 granin genes which are displayed as a portion of the total, with each granin expression level shown in a color-coded manner. Granin expression is indicated as RPKM in the 16 brain regions, consisting of **(a)** amygdaloid complex, **(b)** anterior cingulate cortex, **(c)** cerebellar cortex, **(d)** dorsolateral prefrontal cortex, **(e)** hippocampus, **(f)** inferolateral temporal cortex, **(g)** mediodorsal nucleus of thalamus, **(h)** orbital frontal cortex, **(i)** posterior superior temporal cortex, **(j)** posteroventral parietal cortex, **(k)** primary auditory cortex, **(l)** primary motor cortex, **(m)** primary somatosensory cortex, **(n)** primary visual cortex, **(o)** striatum, and **(p)** ventrolateral prefrontal cortex. Six development periods were studied consisting of (1) early prenatal, (2) late prenatal, (3) infancy, (4) childhood, (5) adolescence, and (6) young adult (shown as stages #1-6). Bar graphs show the total granin gene expression values as the average RPKM +/- s.e.m., with statistical significance (*p < 0.05, **p < 0.01, ***p < 0.001, and ****p < 0.0001) assessed by two-way ANOVA followed by Tukey’s post hoc multiple comparisons test.

Total granin gene expression was evaluated as the sum of each of the granin gene expression levels for six developmental stages from prenatal to infancy, childhood, adolescence, and young adult (Figure 3). The 16 human brain regions studied consisted of the amygdaloid complex, anterior cingulate cortex, cerebellar cortex, dorsolateral prefrontal cortex, hippocampus, inferolateral temporal cortex, mediodorsal nucleus of thalamus, orbital frontal cortex, posterior superior temporal cortex, posteroventral parietal cortex, primary auditory cortex, primary motor cortex, primary somatosensory cortex, primary visual cortex, striatum, and ventrolateral prefrontal cortex.

The majority of the brain regions (14 regions shown in panels a-b, d-n, and p of Figure 3) displayed significant differences in total granin expression among the developmental stages from prenatal through young adult stages. A similar feature among these 14 brain regions was the finding that total granin expression increased from prenatal (early and late prenatal) to infancy stages, followed by a drop at the childhood compared to the infant stage, and then an increase was observed from childhood to adolescence and adult stages. These data suggest that increased granin gene expression from prenatal to infancy may represent the functional importance of these secretory vesicle proteins in brain development. Moreover, a second phase of increased total granin expression during childhood to adolescence to young adult stages (for brain regions shown in panels b, d, e-j, m, n, and p of Figure 3) may reflect involvement of granins in these regions for development during childhood to young adult.

However, two brain regions, striatum and cerebellar cortex, showed minimal changes in total granin expression during development. The striatum (panel o, Figure 3) displayed small differences in total granin expression at the early prenatal compared to infancy stage, and infancy compared to childhood and adolescent stages. The cerebellar cortex (panel c, Figure 3) showed no changes in total granin expression among the developmental stages.

These data also show that expression of the granin genes is maintained throughout development in the 16 human brain regions studied.

### Relative expression of each granin gene during development

To evaluate the relative expression levels of each of the granins among the 16 brain regions at the 6 developmental stages, the percent of total granin expression was calculated for each granin and summarized (Table 3) based on detailed expression data in the brain regions among the developmental periods (Supplementary Table S1). The 8 granin genes each showed 3–20% levels of expression of the total granin expression levels. *VGF*, *CHGA*, and *SCG2* genes displayed expression levels of 3–4 to 12–17% of total granins. Compared to all granins, *VGF*, *CHGA*, and *SCG2* showed the lowest level of expression in several brain regions, including primary motor cortex and primary somatosensory cortex. *CHGB* and *SCG3* showed a range of expression of 9–20% of total granins; *CHGB* showed the highest level of expression in cerebellar cortex at the childhood to young adult stages. *SCG5*, *proSAAS*, and *GNAS* displayed expression at 12–19% of total granins; high level expression of these granins occurred mainly in the prenatal stage. These data show the ranges of granins expressed during development among multiple brain regions.

**Table 3:** Ranges of granin gene expression during human brain development.

Granin gene	Range of expression during development in 16 human brain regions
*VGF*	4–12%
*CHGA*	3–14%
*CHGB*	9–20%
*SCG2*	3–17%
*SCG3*	10–17%
*SCG5*	12–17%
*PCSK1N* (proSAAS)	13–18%
*GNAS* (NESP55)	12–19%

The range of expression values for each granin gene, among the 16 brain regions in 6 developmental periods (Supplementary Table S1) is shown as the percent of total granin gene expression.

### Regulation of *VGF*, *SCG2*, and *CHGA* gene expression during development in human brain

The expression levels of the *VGF*, *SCG2,* and *CHGA* genes were each assessed for developmental regulation in the 16 brain regions. Significant regulation of these granin genes was observed during development.

**Figure 4:**
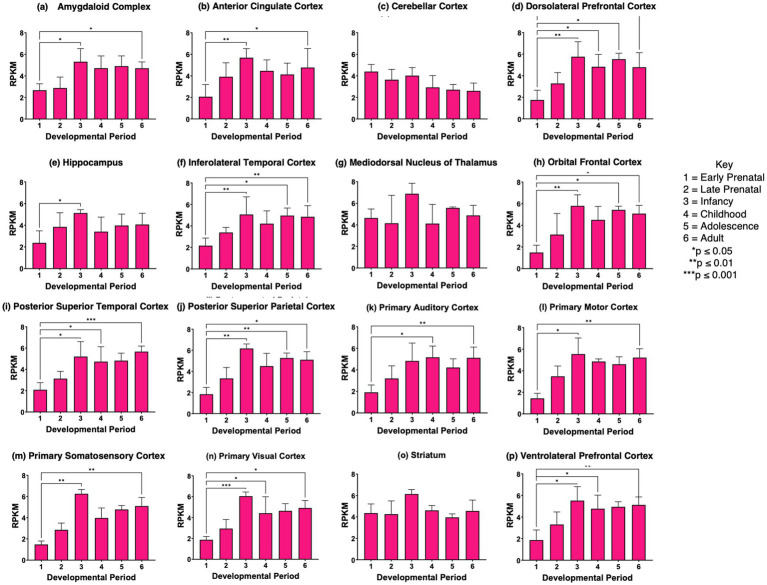
*VGF* gene expression during development in human brain. Expression levels of the *VGF* gene in 16 brain regions are shown for **(a)** amygdaloid complex, **(b)** anterior cingulate cortex, **(c)** cerebellar cortex, **(d)** dorsolateral prefrontal cortex, **(e)** hippocampus, **(f)** inferolateral temporal cortex, **(g)** mediodorsal nucleus of thalamus, **(h)** orbital frontal cortex, **(i)** posterior superior temporal cortex, **(j)** posteroventral parietal cortex, **(k)** primary auditory cortex, **(l)** primary motor cortex, **(m)** primary somatosensory cortex, **(n)** primary visual cortex, **(o)** striatum, and **(p)** ventrolateral prefrontal cortex. Six development periods were studied consisting of (1) early prenatal, (2) late prenatal, (3) infancy, (4) childhood, (5) adolescence, and (6) young adult. Graphs show the average RPKM +/- s.e.m. for VGF expression, with statistical significance of (*p<0.05, **p<0.01, and ***p<0.001) assessed by Kruskal-Wallis non-parametric test followed by Dunn’s multiple comparison post hoc test.

*VGF* gene expression was significantly regulated during development in 12 of the 16 brain regions examined, shown in panels a-b, d-f, h-j, l-n, and p of Figure 4. VGF in these brain regions showed significant increases from the prenatal phase to the infancy stage. While this increase was observed in the primary auditory cortex, the values did not reach significance. During the stages of childhood to adolescence and young adult, no significant increase in *VGF* expression was observed. The young adult stage showed higher *VGF* expression than the prenatal stage in most brain regions (panels a, b, d, f, h-n, and p of Figure 4). These data demonstrate significant regulation of *VGF* during prenatal to infancy stages among the majority of brain regions studied.

**Figure 5:**
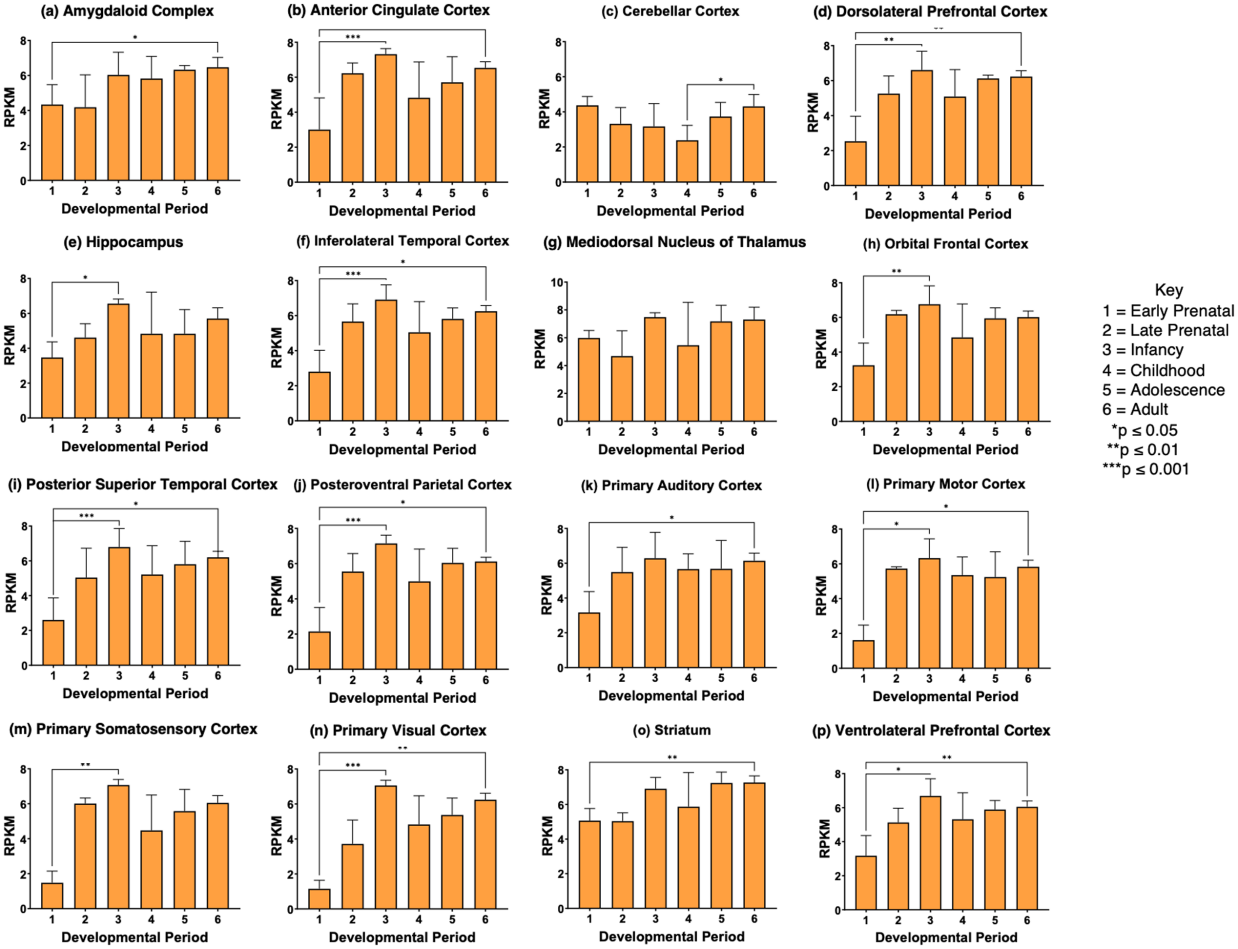
*SCG2* gene expression during development in human brain. Expression levels of the *SCG2* gene in 16 brain regions are shown for **(a)** amygdaloid complex, **(b)** anterior cingulate cortex, **(c)** cerebellar cortex, **(d)** dorsolateral prefrontal cortex, **(e)** hippocampus, **(f)** inferolateral temporal cortex, **(g)** mediodorsal nucleus of thalamus, **(h)** orbital frontal cortex, **(i)** posterior superior temporal cortex, **(j)** posteroventral parietal cortex, **(k)** primary auditory cortex, **(l)** primary motor cortex, **(m)** primary somatosensory cortex, **(n)** primary visual cortex, **(o)** striatum, and **(p)** ventrolateral prefrontal cortex. Six development periods were studied consisting of (1) early prenatal, (2) late prenatal, (3) infancy, (4) childhood, (5) adolescence, and (6) young adult. Graphs show the average RPKM +/- s.e.m. for *SCG2* expression, with statistical significance of (*p<0.05, **p<0.01, and ***p<0.001) assessed by Kruskal-Wallis non-parametric test followed by Dunn’s multiple comparison post hoc test.

*SCG2* gene expression was significantly elevated during development from prenatal to infancy stages in 11 of the 16 regions (panels b, d, e, f, h-j, l, m, n, p of Figure 5). *SCG2* showed higher expression at the young adult stage compared to the prenatal stage for 11 brain regions shown in panels a, b, d, f, i-l, m, n-p of Figure 5. These data show that in several brain regions, *SCG2* expression increases from prenatal to infancy stages, as found for *VGF* in numerous brain regions. The increase in *SCG2* expression at the young adult compared to the prenatal stage differs from *VGF* or *CHGA* changes during brain development.

**Figure 6:**
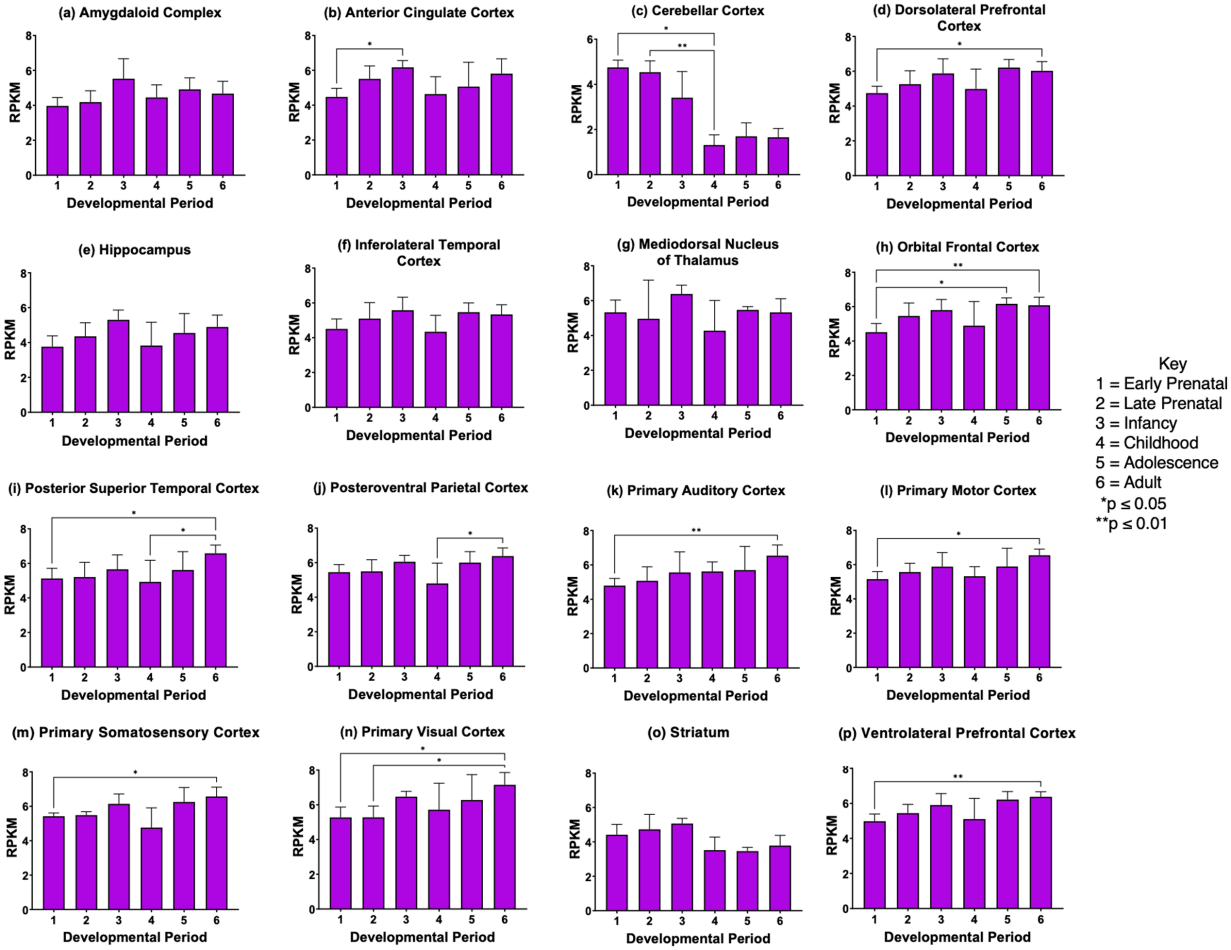
*CHGA* gene expression during development in human brain. Expression levels of the *CHGA* gene in 16 brain regions are shown for **(a)** amygdaloid complex, **(b)** anterior cingulate cortex, **(c)** cerebellar cortex, **(d)** dorsolateral prefrontal cortex, **(e)** hippocampus, **(f)** inferolateral temporal cortex, **(g)** mediodorsal nucleus of thalamus, **(h)** orbital frontal cortex, **(i)** posterior superior temporal cortex, **(j)** posteroventral parietal cortex, **(k)** primary auditory cortex, **(l)** primary motor cortex, **(m)** primary somatosensory cortex, **(n)** primary visual cortex, **(o)** striatum, and **(p)** ventrolateral prefrontal cortex. Six development periods were studied consisting of (1) early prenatal, (2) late prenatal, (3) infancy, (4) childhood, (5) adolescence, and (6) young adult. Graphs show the average RPKM +/- s.e.m. for *SCG2* expression, with statistical significance of (*p<0.05 and **p<0.01) assessed by Kruskal-Wallis non-parametric test followed by Dunn’s multiple comparison post hoc test.

*CHGA* gene expression showed significant increases in *CHGA* expression at the adolescence or young adult stages compared to the prenatal stages in several brain regions (panels d, h, i, k-n, and p of Figure 6). Also, cerebellar cortex *CHGA* expression at the prenatal stages (early and late prenatal) became decreased in the childhood stage by more than 75%; this decrease was not observed in other brain regions. These data show differences in the regulation of *CHGA* compared to *VGF* and *SCG2* expression during development in human brain.

## Modest regulation of *CHGB* and *SCG3* genes, and minimal changes in *SCG5*, *PCSK1N*, and *GNAS* granin genes during brain development

**Figure 7:**
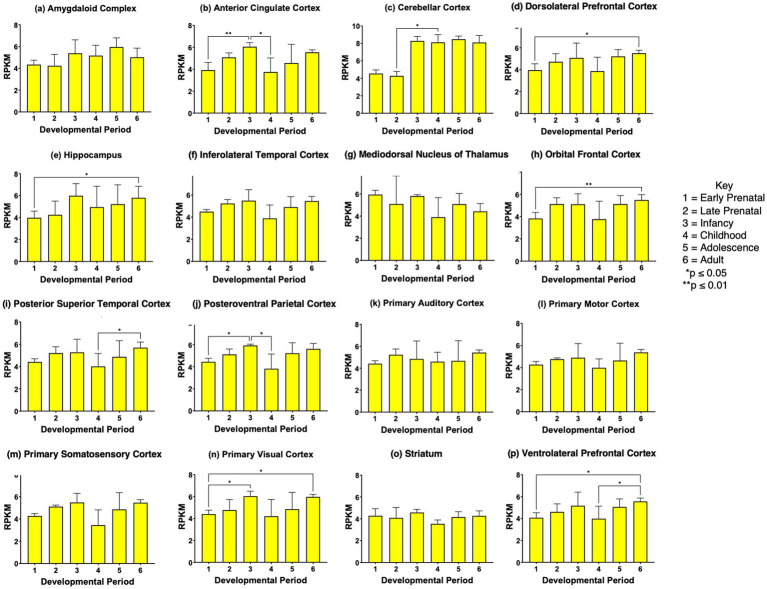
*CHGB* gene expression during development in human brain. Expression levels of the *CHGB* gene in 16 brain regions are shown for **(a)** amygdaloid complex, **(b)** anterior cingulate cortex, **(c)** cerebellar cortex, **(d)** dorsolateral prefrontal cortex, **(e)** hippocampus, **(f)** inferolateral temporal cortex, **(g)** mediodorsal nucleus of thalamus, **(h)** orbital frontal cortex, **(i)** posterior superior temporal cortex, **(j)** posteroventral parietal cortex, **(k)** primary auditory cortex, **(l)** primary motor cortex, **(m)** primary somatosensory cortex, **(n)** primary visual cortex, **(o)** striatum, and **(p)** ventrolateral prefrontal cortex. Six development periods were studied consisting of (1) early prenatal, (2) late prenatal, (3) infancy, (4) childhood, (5) adolescence, and (6) young adult. Graphs show the average RPKM +/- s.e.m. for *CHGB* expression, with statistical significance of (*p<0.05, **p<0.01, and ***p<0.001) assessed by Kruskal-Wallis non-parametric test followed by Dunn’s multiple comparison post hoc test.

**Figure 8:**
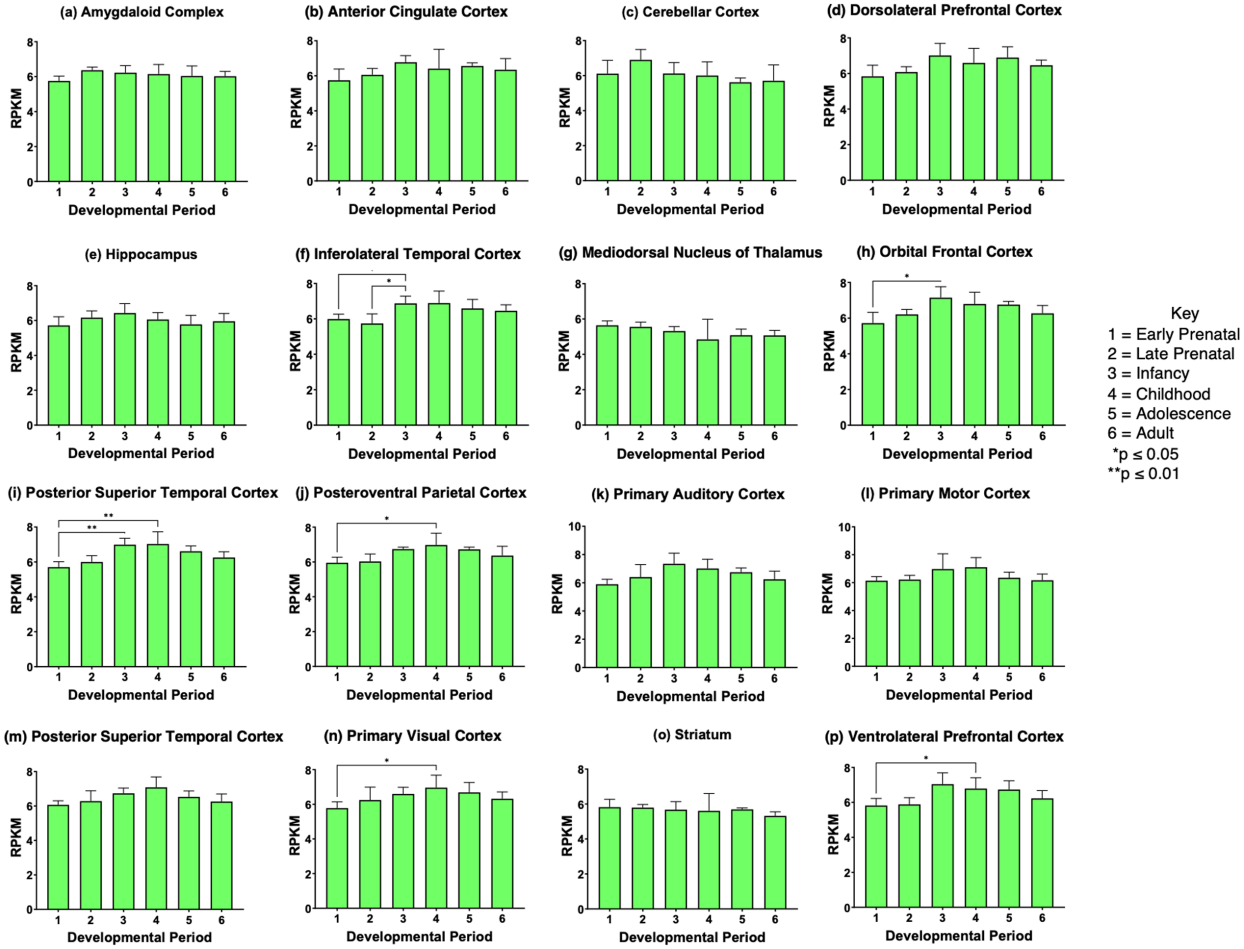
*SCG3* gene expression during development in human brain. Expression levels of the *SCG3* gene in 16 brain regions are shown for **(a)** amygdaloid complex, **(b)** anterior cingulate cortex, **(c)** cerebellar cortex, **(d)** dorsolateral prefrontal cortex, **(e)** hippocampus, **(f)** inferolateral temporal cortex, **(g)** mediodorsal nucleus of thalamus, **(h)** orbital frontal cortex, **(i)** posterior superior temporal cortex, **(j)** posteroventral parietal cortex, **(k)** primary auditory cortex, **(l)** primary motor cortex, **(m)** primary somatosensory cortex, **(n)** primary visual cortex, **(o)** striatum, and **(p)** ventrolateral prefrontal cortex. Six development periods were studied consisting of (1) early prenatal, (2) late prenatal, (3) infancy, (4) childhood, (5) adolescence, and (6) young adult. Graphs show the average RPKM +/- s.e.m. for *SCG3* expression, with statistical significance of (*p<0.05, **p<0.01, and ***p<0.001) assessed by Kruskal-Wallis non-parametric test followed by Dunn’s multiple comparison post hoc test.

The *CHGB* and *SCG3* genes showed modest upregulation in several brain regions comparing prenatal to postnatal stages of infancy, childhood, adolescence, and adult (Figures 7 and 8). However, the *SCG5*, *proSAAS*, and *GNAS* genes showed essentially no regulation during development (Supplementary Figures S1–S3). These findings indicate differential regulation of granin genes in human brain during development.

## Overall findings: differential upregulation of *VGF*, *SCG2*, *CHGA*, *CHGB*, and *SCG3* granin genes during human brain development

**Figure 9:**
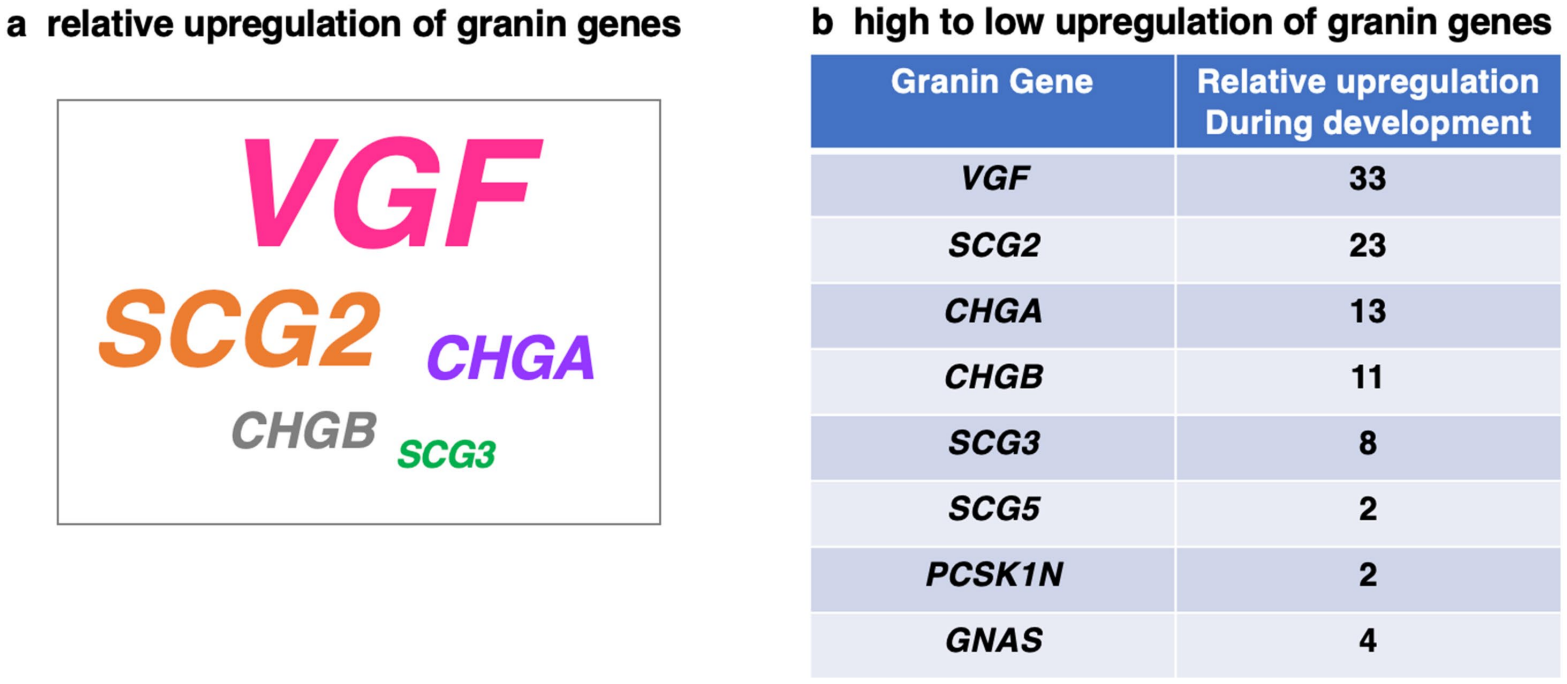
Summary of differential upregulation of *VGF*, *SCG2*, *CHGA*, *CHGB*, and *SCG3* granin genes during human brain development. The relative upregulation of these granin genes is illustrated by the size of the gene name **(a)** based on the number of significant comparisons of upregulation from the prenatal periods to the postnatal periods **(b)**. *VGF*, *SCG2*, *CHGA*, *CHGB*, and *SCG3* showed significant upregulation during development in human brain **(a)**. In contrast, the other granin genes of *SCG5*, *PCSK1N*, and *GNAS* showed almost no developmental regulation **(b)**.

A schematic illustration (Figure 9A) shows the relative prevalence of significant upregulation of the *VGF*, *SCG2*, *CHGA*, *CHGB*, and *SCG3* granin genes in 16 brain regions during transition from prenatal to postnatal periods of infancy, childhood, adolescence, and adult. The relative upregulation of these granin genes was calculated based on the number of significant paired comparisons of upregulation from the prenatal periods to the postnatal periods (Figure 9B). In contrast, the other granin genes of *SCG5*, *PCSK1N*, and *GNAS* showed almost no developmental regulation. *VGF* and *SCG2* genes displayed the greatest prevalence of upregulation in human brain during development.

## Discussion

The granin genes participate in brain cell–cell signaling as neuropeptide transmitters that are essential for brain functions. The goal of this study was to assess the hypothesis that expression of granin genes in human brain is regulated during the developmental stages of prenatal to infancy, childhood, adolescence, and adult. The expression of *VGF* was significantly increased from the prenatal to infancy stages in the anterior cingulate cortex, dorsolateral prefrontal cortex, inferolateral temporal cortex, orbital frontal cortex, posteroventral parietal cortex, primary somatosensory cortex, and primary visual cortex. *SCG2* expression was also significantly increased from the prenatal to infancy stages, observed in the anterior cingulate cortex, dorsolateral prefrontal cortex, inferolateral temporal cortex, orbital frontal cortex, posterior superior temporal cortex, posteroventral parietal cortex, primary somatosensory cortex, and primary visual cortex. *CHGA* expression was more abundant at the young adult age compared to the prenatal stage. *CHGB* and *SCG3* displayed minor changes in gene expression levels during development. The other *SCG5*, *PCSK1N*, and *GNAS* genes showed primarily no changes in expression during development of the human brain in the 16 regions studied. The overall relative upregulation of *VGF*, *SCG2*, with lesser upregulation of *CHGA*, *CHGB*, and *SCG3*, is schematically shown in Figure 9. These findings demonstrate that *VGF* and *SCG2* display the greatest extent of upregulation among the granin genes from prenatal to postnatal to adult stages of development.

This study utilized the Allen Human Brain Atlas resource that provides expression data for all human genes in brain (Shen et al., 2012). The data are provided as an open resource to investigators. The developmental expression data, for prenatal to young adult stages, was obtained by RNA-seq analysis for 16 brain regions.

The rationale for investigating granin gene expression regulation during development is based on the important functions of granins in neuronal cell–cell signaling required for brain functions and behaviors. Animal studies of granin gene knockout have demonstrated roles for granins in memory, depression, energy expenditure, tauopathy, angiogenesis, and related (Lin et al., 2015; Watson et al., 2009; Pereda et al., 2015; Jati et al., 2025; Dai et al., 2022). Expression of granins provides diverse neuropeptide transmitters generated from the granin proteins by proteolytic processing, which provides a multitude of diverse neuropeptides. It is estimated that hundreds to thousands of neuropeptides are present in the brain, with novel neuropeptides being discovered in the field with advanced mass spectrometry technology (Hook and Bandeira, 2015; Romanova and Sweedler, 2015; Delaney and Li, 2019; Sauer et al., 2021; Anapindi et al., 2022; Fields et al., 2024).

The hypothesis for the regulation of granin genes during development in human brain regions has been addressed by this study. Results show that expression of selected granin genes are regulated during development from prenatal and infancy stages to childhood, adolescence, and adult periods. Furthermore, the regulation of granin expression occurs in specific brain regions. It will be important to assess the developmental neuropeptides and their biological functions that result from granin gene expression regulation in the multitude of human brain regions from young to adult and older ages.

Human brain disorders display dysregulated granin neuropeptides that include Alzheimer’s disease (Higginbotham et al., 2020; Podvin et al., 2022; Quinn et al., 2023; Haque et al., 2023), Parkinson’s disease (Rotunno et al., 2020), schizophrenia (Takahashi et al., 2006; Podvin et al., 2024), and other neurological and psychiatric disease conditions. It will be important to define the spectrum of regulated neuropeptides participating in human brain diseases to identify novel neuropeptide biomarkers and candidate targets for therapeutics discovery.

Of particular interest is expression of the *VGF* granin which was found by this study to display the largest degree of upregulation compared to the other granin genes during development of the human brain. In Alzheimer’s disease (AD) brain, significant decreases in VGF neuropeptides in the CSF (cerebrospinal fluid) have been found to be associated with cognitive decline (Haque et al., 2023). Furthermore, VGF neuropeptides are dysregulated in in AD brain along with other granin neuropeptides (Higginbotham et al., 2020; Podvin et al., 2022; Quinn et al., 2023; Haque et al., 2023). Overexpression of *VGF* has been shown to result in improved cognitive function (Lin et al., 2021). It will be important to investigate the functions of regulated VGF neuropeptides in AD.

The results of this study provide new insight into the developmental upregulation of the granin genes *VGF*, *SCG2*, *CHGA*, *CHGB*, and *SCG3* during human brain development from prenatal to post-natal periods of infancy, childhood, adolescence, and adult. These data utilized gene expression data of the Allen Human Brain Atlas program that selected the samples from 16 brain regions from 6 developmental periods (Table 1). The numbers of samples per group varied from 4 to 12 samples that included males and females. A limitation of these data are the low numbers of samples per group. It will be beneficial in future studies to utilizes larger group sizes for evaluation of the significant developmental regulation of granin genes in human brain.

In conclusion, this investigation of the developmental regulation of granin genes in human brain demonstrated selected changes in expression of granins during prenatal to infancy and childhood stages, as well as development from childhood to adolescence and adult stages. Granin expression occurs throughout the brain at all stages of development. These findings provide a fundamental understanding of granin gene expression during human brain development that is essential for brain functions and behaviors throughout the decades of life.

## Data Availability

The original contributions presented in the study are included in the article/Supplementary material, further inquiries can be directed to the corresponding author.

## Footnotes

^1^ http://www.brain-map.org/
