# Supplementary material for: Regulation of granin neuropeptide gene expression in human brain during development
Source: Front Mol Neurosci. 2025 Dec 2;18:1666795. doi: 10.3389/fnmol.2025.1666795 (PMC12706669; doi:10.3389/fnmol.2025.1666795)
Supplement: Supplementary file 2 [file Presentation_1.pdf]

Supplementary Materials

Regulation of Granin Neuropeptide Gene Expression in Human Brain during Development

Laura L. Demsey,[1] Sonia Podvin,[1] and Vivian Hook[1,2*]

[1]Skaggs School of Pharmacy and Pharmaceutical Sciences, University of California, San Diego, La Jolla, CA 92093;
[2]Department of Neurosciences and Department of Pharmacology, School of Medicine, University of California, San Diego, La Jolla, CA 92093

Figure S1. ***SCG5*** **gene expression during development in human brain**
Expression levels of the *SCG5* gene in 16 brain regions shown are panels a-p, for 6 development periods of (1) early prenatal, (2) late prenatal, (3) infancy, (4) childhood, (5) adolescence, and (6) young adult. Graphs show the average RPKM + s.e.m. for *SCG5* expression, with statistical significance of (*p≤0.05, **p≤0.01, and ***p≤0.001) assessed by Kruskal-Wallis non-parametric test followed by Dunn's multiple comparison post hoc test.

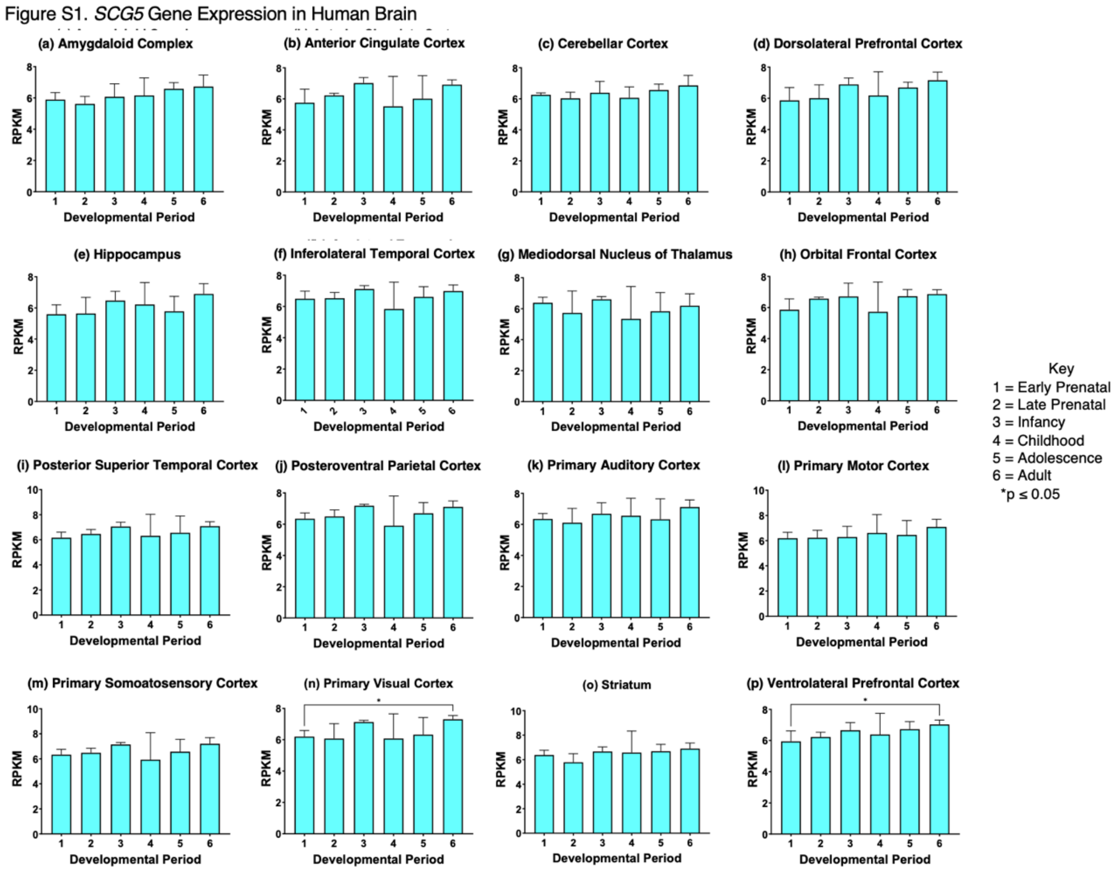

Figure S2. ***PCSK1N*** **(proSAAS) gene expression during development in human brain**
Expression levels of the *PCSK1N* gene in 16 brain regions shown are panels a-p, for 6 development periods of (1) early prenatal, (2) late prenatal, (3) infancy, (4) childhood, (5) adolescence, and (6) young adult. Graphs show the average RPKM $\pm$ s.e.m. for *PCSK1N* expression, with statistical significance of (*$p \leq 0.05$, **$p \leq 0.01$, and ***$p \leq 0.001$) assessed by Kruskal-Wallis non-parametric test followed by Dunn's multiple comparison post hoc test.

Figure S2. *PCSK1N* (proSAAS) Gene Expression in Human Brain

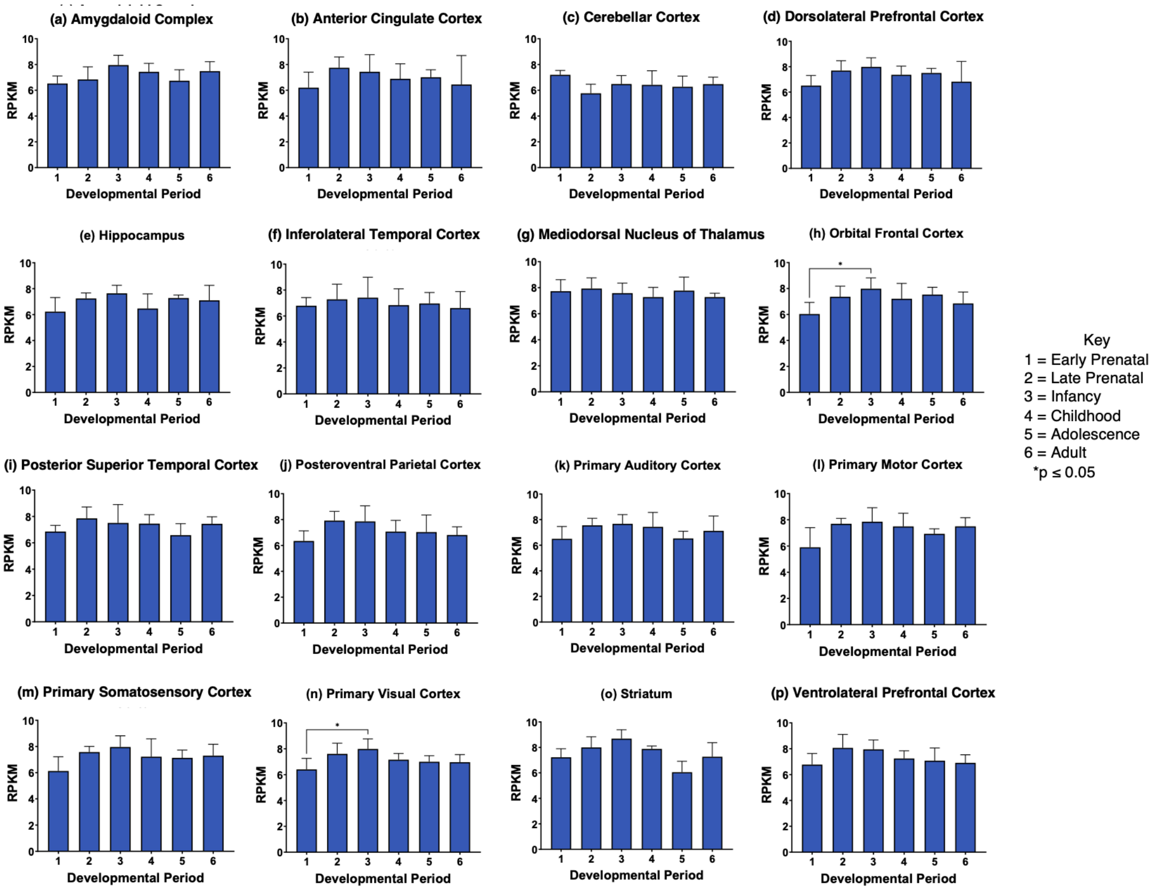

Figure S3. ***GNAS*** **(NESP55) gene expression during development in human brain**
Expression levels of the *GNAS* gene in 16 brain regions shown are panels a-p, for 6 development periods of (1) early prenatal, (2) late prenatal, (3) infancy, (4) childhood, (5) adolescence, and (6) young adult. Graphs show the average RPKM + s.e.m. for *GNAS* expression, with statistical significance of (*p≤0.05, **p≤0.01, and ***p≤0.001) assessed by Kruskal-Wallis non-parametric test followed by Dunn's multiple comparison post hoc test.

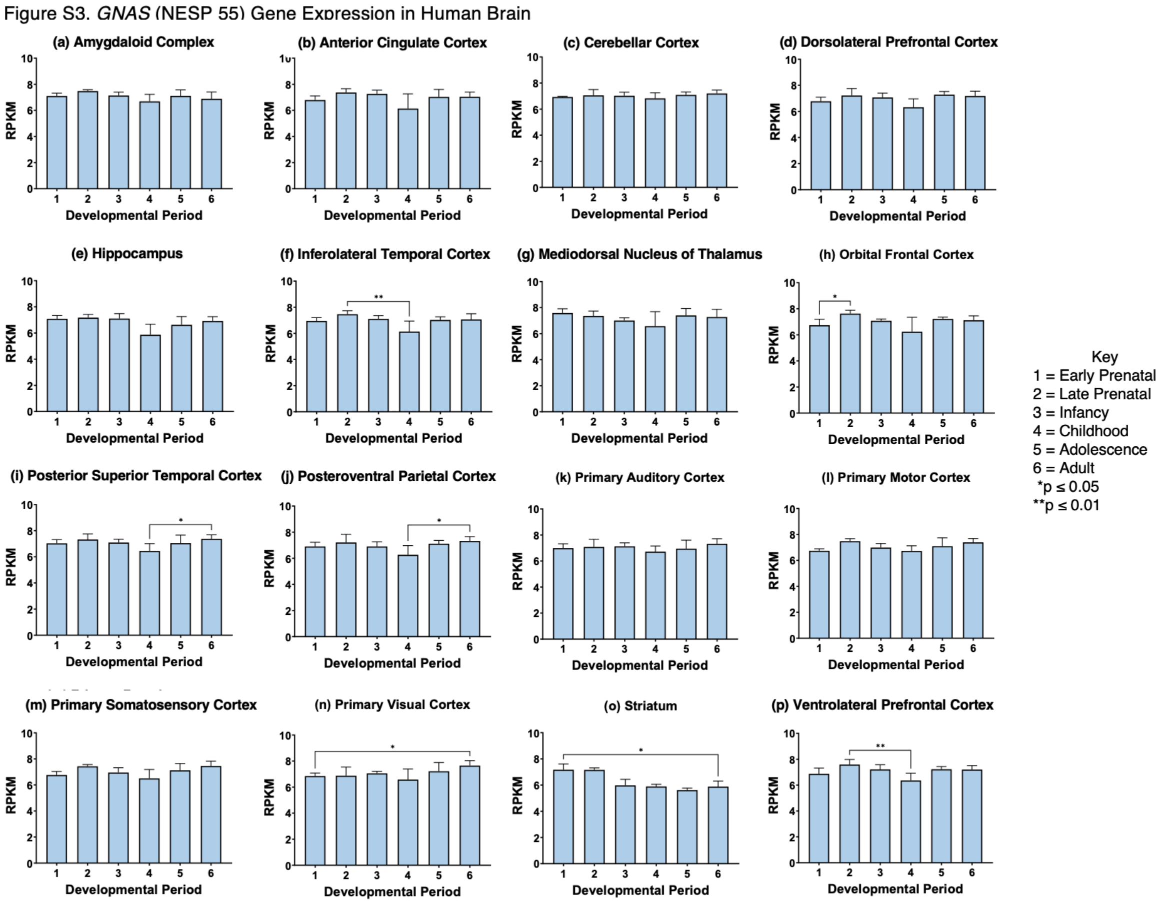

Supplemental Table S1 (see excel file)

Granin gene expression values in human brain regions by developmental period

Gene expression values for each granin gene in 16 human brain regions is shown for 6 development periods of early prenatal, late prenatal, infancy, childhood, adolescence, and adult. This table is provided as a separate excel file.
